# Comparative survey data on sociodemographic predictors of diversity tolerance among selected university students in Ghana and South Africa

**DOI:** 10.1016/j.dib.2021.106771

**Published:** 2021-01-17

**Authors:** Elizabeth Biney, Olusegun Sunday Ewemooje, Acheampong Yaw Amoateng

**Affiliations:** a Population and Health Research Entity, Faculty of Humanities, North-West University (Mafikeng Campus), North West, South Africa; b Department of Statistics, Federal University of Technology Akure, Akure, Nigeria

**Keywords:** Diversity, Tolerance, Higher education, Multiculturalism, Sub-Saharan Africa, Youth

## Abstract

This article presents an extensive comparison of survey data on tolerance attitudes of 1758 participants from two public universities in sub-Saharan Africa, the University of Ghana and North-West University. Multi-stage and other sampling procedures were employed to collect the data between 2016 and 2017. Data were analysed using frequencies, percentages and cross-tabulations for each institution separately. Overall, participants expressed a high level of tolerance to others of different racial and ethnic backgrounds, albeit higher for those in the University of Ghana than North-West University. The findings further revealed that participants’ gender, academic level, family socioeconomic status, and parental educational level were significantly associated with tolerant attitudes, depending on the educational institution.

## Specifications Table

**Table:** 

Subject	Social Sciences, Humanities.
Specific subject area	Demography, Education, Population Studies, Sociology, Youth Studies
Type of data	Tables
How data were acquired	Information was gathered by administering semi-structured questionnaires to sampled students. A copy of the questionnaire is attached as a supplementary file.
Data format	Raw
	Analysed
Parameters for data collection	Information collected included individual demographic and socioeconomic characteristics, family background, reported attitudes toward foreigners and cultural diversity (see supplementary files).
Description of data collection	Data were obtained from 912 to 846 students from the University of Ghana and North-West University respectively, using a multi-stage sampling (both stratified and systematic sampling) technique.
Data source location	The University of Ghana, Greater Accra Region, Ghana. North-West University, Mafikeng, Vaal Triangle, Potchefstroom, South Africa.
Data accessibility	Data is included in this article

## Value of the Data

• Africa is often characterized as a region of rampant intolerance, division and conflict in an increasingly globalized world—whether that tensions be based on ethnicity, religion, nationality, or sexual orientation [1]. This negative image persists despite a paucity of data gaging attitudes towards diversity in the African context. The dataset aims to contribute to the knowledge base and broader debates on tolerance in this regard.
• Respect for and acceptance of diversity is crucial in maintaining social cohesion in a population of different cultures, races/ethnicities, and religions [2,3]. The dataset provides vital information on individual background characteristics, parental characteristics and self-reported attitudes toward ethno-racial differences and human diversity (see supplementary file). This can be useful for anyone who has an empirical interest in multiculturalism or interculturality, intergroup relations and group conflict, particularly in the youth population or higher education sector.
• Higher education is posited as promoting a more enlightened and cosmopolitan worldview, attenuating prejudice, and fostering intergroup tolerance [4], [5], [6]. Thus, people participating in the higher education system are exposed to such modernizing values as good citizenship and inclusivity needed for positive social relations [3]. The data, therefore, can be used to test the liberalizing attitudinal effects of education— higher education is associated with more favorable attitudes toward different cultures or groups.
• The datasets are primarily focused on two institutions of diverse social contexts in sub-Saharan Africa: The University of Ghana is the premier and largest public university in Ghana, a multi-ethnic and relatively harmonious society; whereas North-West University is a unified multi-campus public university in South Africa, a polarized multiracial society. Given both sample sites are culturally diverse environments, the dataset can be used in descriptive or comparative research into how diverse social backgrounds (educational, racial/ethnic, class, religion and gender) shape individual attitude towards and or acceptance of diversity.

## 1 Data Description

Table 1 summarises sample demographic and socioeconomic characteristics for the two groups. Overall, females constituted more than half of the total sample in both universities – University of Ghana (51.8%) and North-West University (54.8%). The average age of participant from the University of Ghana was 21.1 years old, whereas that of north-West University was 21.3 years old. The ethnic breakdown of the sample shows that more than half (58.8%) of participants from the University of Ghana were Akan, while black Africans constituted more than three-quarters (88.2%) of participants in North-West University. The majority of participants in both the University of Ghana (92.3%) and North-West University (86.5%) reported Christian religious affiliation. More than half (56.4%) of the participants in the University of Ghana were in their intermediate year of academic study, whereas more than a third of participants in North-West University were in their first and final years of study. More than two-thirds (69.4%) of participants in North-West University reported belonging to middle-wealth households, whereas more than two-fifths of participants from the University of Ghana reported belonging to middle-wealth (48.4%) or richer (45%) households. Breakdown by parental education shows that more than half (56.4%) of participants from the University of Ghana had fathers with tertiary level education compared to 44.7% of participants from North-West University. Finally, more than a third of participants from the University of Ghana had mothers with secondary-level education or higher, while more than two-fifths of participants from North-West University had mothers with secondary-level education (45%) or higher (43.4%).

**Table 1:** Distribution of sample background characteristics.

	University of Ghana		North-West University	
	Frequency	Percent	Frequency	Percent
Age – mean/sd	21.1/3.08		21.3/2.44	
Gender				
Male	439	48.2	382	45.2
Female	471	51.8	464	54.8
Ethnicity/Race				
Akan	530	58.8		
Ga-Adangbe	109	12.1		
Ewe	140	15.5		
Other	123	13.6		
Black African			733	88.2
White			61	7.3
Coloured			30	3.7
Indian/Asian			7	0.8
Religion				
Christian	772	92.3	642	86.5
Muslim	44	5.3	12	1.6
Other	20	2.4	88	11.9
Academic Level				
First year	156	17.1	294	34.8
Intermediate year	514	56.4	231	27.3
Final year	242	26.5	321	37.9
Father's Education				
Below Secondary	175	20.6	212	29.7
Secondary	196	23.0	183	25.6
Tertiary	480	56.4	319	44.7
Mother's Education				
Below Secondary	224	25.3	95	11.6
Secondary	349	39.3	368	45.0
Tertiary	314	35.4	355	43.4
Family SES				
Poorer	59	6.6	167	19.9
Middle	434	48.4	583	69.4
Richer	404	45.0	90	10.7
Total	912	100.0	846	100.0

**Fig. 1:**
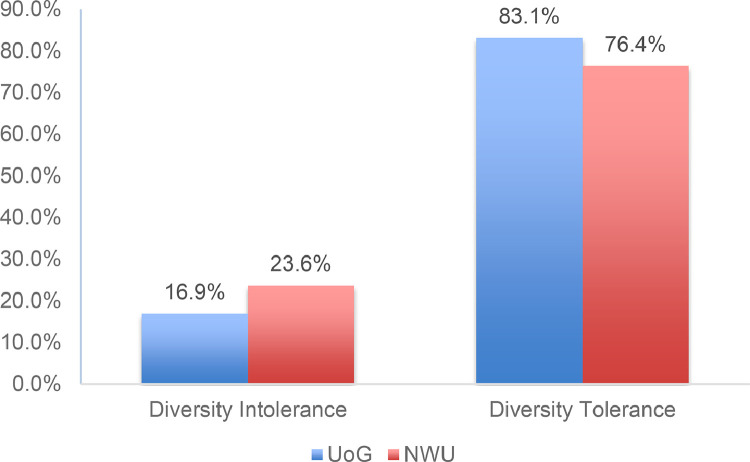
Tolerance to diversity by educational institution.

Fig. 1 shows the tolerance level of students from University of Ghana and North-West University towards members of different racial/ethnic groups; Tables 2 and 3 show the distribution of the responses to the eight items measuring tolerance of diversity. As Fig. 1 shows, on the whole, the level of tolerance or openness to diversity was rather high for both groups, although students in the University of Ghana more frequently (83.1%) indicated tolerance towards people of different race/ethnicity than those in North-West University (76.4%). Further, the majority of students from both Ghana and South Africa indicated that they were open to interacting with people from different race/ethnicity to themselves to a large extent or a very large extent. Similarly, as Table 3 shows, the mean score for students’ tolerance to diversity was significantly higher than the scale midpoint for both University of Ghana and North-West University at different levels. Table 4 shows the relationship between the eight items measuring tolerance of diversity. The students’ attitudes were found to be significant positive correlates of tolerance—ranging from 0.429 to 0.892 for the University of Ghana and 0.283 to 0.881 for the North-West University—indicating that students at the University of Ghana were more tolerant of diversity than counterparts at the North-West University.

**Table 2:** Percentage distribution of tolerance of diversity in the University of Ghana and North-West University.

	University of Ghana						North-West University					
	No extent	Small extent	Medium extent	Large extent	Very large extent	Total	No extent	Small extent	Medium extent	Large extent	Very large extent	Total
Attending lectures with someone of a different ethnic/race group	5.5%	3.0%	12.6%	30.1%	48.8%	906	4.5%	3.6%	20.4%	24.3%	47.2%	830
Participating in a study group with someone from another ethnic/race group	4.6%	4.3%	15.6%	30.3%	45.2%	898	5.3%	5.8%	21.1%	26.9%	40.9%	829
Sharing accommodation but not the same room	7.8%	7.6%	20.3%	28.8%	35.4%	895	9.4%	8.4%	23.6%	21.7%	36.9%	821
Sharing a room with someone from another ethnic/race group	7.3%	7.7%	22.3%	28.7%	34.0%	901	24.0%	13.9%	25.1%	17.1%	19.9%	818
Being friends with someone from another ethnic/race group	4.6%	3.6%	16.4%	32.9%	42.6%	901	4.7%	4.2%	21.5%	27.5%	42.0%	824
dating someone from another ethnic/race group	7.2%	9.9%	23.7%	26.9%	32.3%	899	14.4%	8.4%	22.9%	21.7%	32.6%	825
socializing with people from another ethnic/race group	4.0%	2.8%	14.4%	31.4%	47.3%	900	4.7%	3.9%	18.1%	25.3%	48.0%	823
Having friends who are members of a different ethnic/race group	3.9%	3.1%	11.2%	32.0%	49.8%	903	5.4%	4.1%	16.2%	26.0%	48.3%	831

**Table 3:** Mean distribution of tolerance of diversity in the University of Ghana and North-West University.

	University of Ghana			North-West University		
	Mean	Std. Deviation	Level of Tolerance	Mean	Std. Deviation	Level of Tolerance
Attending lectures	4.14	1.101	High Tolerance	4.06	1.104	High Tolerance
Participating in a study group	4.07	1.090	High Tolerance	3.92	1.151	Moderate Tolerance
Sharing accommodation but not the same room	3.76	1.229	Moderate Tolerance	3.68	1.299	Moderate Tolerance
Sharing a room	3.74	1.210	Moderate Tolerance	2.95	1.437	Low Tolerance
Being friends	4.05	1.068	High Tolerance	3.98	1.110	Moderate Tolerance
dating	3.67	1.225	moderate tolerance	3.50	1.392	moderate tolerance
socializing with people from another ethnic group	4.15	1.033	high tolerance	4.08	1.113	high tolerance
Having friends who are members of a different ethnic group	4.21	1.020	High Tolerance	4.08	1.137	High Tolerance

**Table 4:** Intercorrelations between participants’ attitudes and diversity tolerance.

	University of Ghana								North-West University								
	1	2	3	4	5	6	7	8	1	2	3	4	5	6	7	8	
1	Attending lectures	1	0.795^⁎⁎^	0.633^⁎⁎^	0.555^⁎⁎^	0.677^⁎⁎^	0.434^⁎⁎^	0.725^⁎⁎^	0.728^⁎⁎^	1	0.724^⁎⁎^	0.522^⁎⁎^	0.283^⁎⁎^	0.612^⁎⁎^	0.336^⁎⁎^	0.641^⁎⁎^	0.638^⁎⁎^
2	Participating in a study group	0.795^⁎⁎^	1	0.687^⁎⁎^	0.612^⁎⁎^	0.708^⁎⁎^	0.447^⁎⁎^	0.697^⁎⁎^	0.690^⁎⁎^	0.724^⁎⁎^	1	0.573^⁎⁎^	0.388^⁎⁎^	0.537^⁎⁎^	0.292^⁎⁎^	0.564^⁎⁎^	0.555^⁎⁎^
3	Sharing accommodation but not the same room	0.633^⁎⁎^	0.687^⁎⁎^	1	0.705^⁎⁎^	0.604^⁎⁎^	0.429^⁎⁎^	0.593^⁎⁎^	0.600^⁎⁎^	0.522^⁎⁎^	0.573^⁎⁎^	1	0.470^⁎⁎^	0.505^⁎⁎^	0.346^⁎⁎^	0.495^⁎⁎^	0.483^⁎⁎^
4	Sharing a room	0.555^⁎⁎^	0.612^⁎⁎^	0.705^⁎⁎^	1	0.646^⁎⁎^	0.465^⁎⁎^	0.581^⁎⁎^	0.562^⁎⁎^	0.283^⁎⁎^	0.388^⁎⁎^	0.470^⁎⁎^	1	0.351^⁎⁎^	0.331^⁎⁎^	0.296^⁎⁎^	0.315^⁎⁎^
5	Being friends	0.677^⁎⁎^	0.708^⁎⁎^	0.604^⁎⁎^	0.646^⁎⁎^	1	0.500^⁎⁎^	0.738^⁎⁎^	0.743^⁎⁎^	0.612^⁎⁎^	0.537^⁎⁎^	0.505^⁎⁎^	0.351^⁎⁎^	1	0.513^⁎⁎^	0.734^⁎⁎^	0.719^⁎⁎^
6	Dating	0.434^⁎⁎^	0.447^⁎⁎^	0.429^⁎⁎^	0.465^⁎⁎^	0.500^⁎⁎^	1	0.525^⁎⁎^	0.485^⁎⁎^	0.336^⁎⁎^	0.292^⁎⁎^	0.346^⁎⁎^	0.331^⁎⁎^	0.513^⁎⁎^	1	0.466^⁎⁎^	0.451^⁎⁎^
7	socializing with people from another ethnic group	0.725^⁎⁎^	0.697^⁎⁎^	0.593^⁎⁎^	0.581^⁎⁎^	0.738^⁎⁎^	0.525^⁎⁎^	1	0.892^⁎⁎^	0.641^⁎⁎^	0.564^⁎⁎^	0.495^⁎⁎^	0.296^⁎⁎^	0.734^⁎⁎^	0.466^⁎⁎^	1	0.881^⁎⁎^
8	Having friends who are members of a different ethnic group	0.728^⁎⁎^	0.690^⁎⁎^	0.600^⁎⁎^	0.562^⁎⁎^	0.743^⁎⁎^	0.485^⁎⁎^	0.892^⁎⁎^	1	0.638^⁎⁎^	0.555^⁎⁎^	0.483^⁎⁎^	0.315^⁎⁎^	0.719^⁎⁎^	0.451^⁎⁎^	0.881^⁎⁎^	1

Table 5 presents the relationship between the patterns of distribution of tolerance to diversity and selected socio-demographic factors for students in Ghana and South Africa, respectively. As Table 4 shows, among University of Ghana students, males (85.4%), students of Ga-Adangbe ethnicity (88.1%), those who belonged to ‘other’ religions (90%), those who were in their final year of study (87.2%), those whose fathers had secondary-level education (88.8%) or mothers had below secondary-level education (84.8%), and those from poorer families/households (89.8%) were the most likely to tolerate diversity. Conversely, among North-West University students, females (79.1%), Coloureds (80.0%), Muslims (83.3%), students who were in their first year of study (78.6%), those whose fathers had tertiary education (78.7%) or mothers had secondary-level education (78.5%), and those from richer families/households (82.2%) were the most likely to tolerate diversity. Lastly, the bivariate analyses show that the academic level and paternal education were significant for students at the University of Ghana, whilst gender, family socioeconomic status, and maternal education were significant for students at the North-West University.

**Table 5:** Diversity tolerance and associated sociodemographic factors by educational institution.

	University of Ghana			North-West University		
	Low	High	p-value	Low	High	p-value
Age - mean (sd)	21.0 (3.36)	21.1 (3.08)		21.7 (3.31)	21.3 (2.44)	
Gender (%)			0.069			0.039
Male	64 (14.6%)	375 (85.4%)		103 (27.0%)	279 (73.0%)	
Female	90 (19.1%)	381 (80.9%)		97 (20.9%)	367 (79.1%)	
Ethnicity/Race (%)			0.286			0.949
Akan	99 (18.7%)	431 (81.3%)				
Ga-Adangbe	13 (11.9%)	96 (88.1%)				
Ewe	20 (14.3%)	120 (85.7%)				
Other	21 (17.1%)	102 (82.9%)				
Black African				169 (23.1%)	564 (76.9%)	
White				15 (24.6%)	46 (75.4%)	
Coloured				6 (20.0%)	24 (80.0%)	
Indian/Asian				2 (28.6%)	5 (71.4%)	
Religion (%)			0.385			0.438
Christian	125 (16.2%)	647 (83.8%)		153 (23.8%)	489 (76.2%)	
Muslim	10 (22.7%)	34 (77.3%)		2 (16.7%)	10 (83.3%)	
Other	2 (10.0%)	18 (90.0%)		18 (20.5%)	70 (79.5%)	
Academic Level (%)			0.036			0.361
First year	32 (20.5%)	124 (79.5%)		63 (21.4%)	231 (78.6%)	
Intermediate year	91 (17.7%)	423 (82.3%)		58 (25.1%)	173 (74.9%)	
Final year	31 (12.8%)	211 (87.2%)		79 (24.6%)	242 (75.4%)	
Father's Education (%)			0.030			0.602
Below Secondary	37 (21.1%)	138 (78.9%)		53 (25.0%)	159 (75.0%)	
Secondary	22 (11.2%)	174 (88.8%)		43 (23.5%)	140 (76.5%)	
Tertiary	86 (17.9%)	394 (82.1%)		68 (21.3%)	251 (78.7%)	
Mother's Education (%)			0.709			0.041
Below Secondary	34 (15.2%)	190 (84.8%)		31 (32.6%)	64 (67.4%)	
Secondary	60 (17.2%)	289 (82.8%)		79 (21.5%)	289 (78.5%)	
Tertiary	56 (17.8%)	258 (82.2%)		80 (22.5%)	275 (77.5%)	
Family SES (%)			0.362			0.043
Poorer	6 (10.2%)	53 (89.8%)		48 (28.7%)	119 (71.3%)	
Middle	73 (16.8%)	361 (83.2%)		136 (23.3%)	447 (76.7%)	
Richer	71 (17.6%)	333 (82.4%)		16 (17.8%)	74 (82.2%)	

## 2 Experimental Design, Materials and Methods

The data forms part of an exploratory investigation under the Religion and Positive Youth Development Project, an initiative of the University of Ghana and the North-West University (Mafikeng Campus). Data collection employed multi-stage cluster sampling procedures in selecting the final respondents from the two universities. First, respondents were divided by faculties using a probability sampling method, stratified sampling. This was achieved through proportional allocation to size based on the student population of each faculty. Departments were then selected from each faculty and each department, core courses at all levels (year of study) were selected for inclusion. Consequently, the student population of each faculty determined the proportion of respondents selected for inclusion in the final sample. Individual faculty samples were further disaggregated by year of study; trained field assistants surveyed the number of students in each faculty, department, year of study and gender as determined a priori through the stratified random sampling procedure.

Using the triangulated sampling procedures, a total of 846 and 912 individuals was obtained from North-West University and the University of Ghana, respectively. Trained field assistants administered semi-structured questionnaires in English to participants in the respective institutions. The data collection occurred in two phases: data from North-West University was collected between September 2015 and April 2016, whilst that of University of Ghana took place between September 2016 and March 2017. Participants completed a series of questions related to biographical information (age, gender, religion, nationality, year of study, faculty, marital status), family socioeconomic conditions, parental characteristics, perceptions of immigration and foreign nationals, as well as attitudes towards diversity.

The outcome variable of interest in the present analyses was *tolerance of diversity*. Because tolerance is a complex [2], multidimensional and context-dependent construct rather than a single universal construct, it is conceptualized and operationalized here in several ways. First, it is broadly understood to mean positive social perceptions and behaviors—acceptance of others, such as relating to individuals from different racial, ethnic, and/or cultural backgrounds [7]. Secondly, indicators developed by the Sociology Department at the University of Johannesburg, South Africa (through the Student Communities Project) were adapted for the data landscapes by the researchers, enabling them to measure students’ attitudes and opinions about other people/groups of diverse backgrounds. The original research instrument from which the survey items were adapted was a social distance scale about willingness to accept different degrees of social proximity with members of various groups. The original instrument itself is essentially an adaptation of the Bogardus social distance scale, used to measure attitudes and prejudice, to suit an African landscape. The original scale measures varying degrees of closeness in people towards other members of diverse social, racial or ethnic groups.

Accordingly, the measure of *tolerance of diversity* was computed as a summated score of the following eight items:

• To what extent do you feel comfortable attending lectures with somebody of a different race/ethnic group than your own?
• To what extent do you feel comfortable participating in a study group with somebody of a different race/ethnic group than your own?
• To what extent do you feel comfortable sharing accommodation but not the same room with somebody of a different race/ ethnic group than your own?
• To what extent do you feel comfortable sharing a room with somebody of a different race/ ethnic group than your own?
• To what extent do you feel comfortable being friends with somebody of a different race/ ethnic group than your own?
• To what extent do you feel comfortable dating with somebody of a different race/ ethnic group than your own?
• To what extent do you feel comfortable socializing with people from another racial group with somebody of a different race/ ethnic group than your own?
• To what extent do you feel comfortable having friends who are members of a different racial/ethnic group with somebody of a different race than your own?

All eight items were measured on a five-point Likert scale ranging from 1 = ‘to no extent’ to 5 = ‘to a very large extent’. Higher means indicated greater levels of tolerance and higher willingness to accept differences, and vice versa. The internal consistency of the instrument showed Cronbach's alpha of reliability coefficients of 0.927 for the University of Ghana sample and 0.885 for North-West University, consistent with a previous study that used the questionnaire in a sample of university students in Johannesburg [3]. However, for ease in cross-tabulation in the bivariate analyses, i.e. Table 3, the responses were dichotomized by splitting them at the mean into tolerance and intolerance of diversity. Thus, a score lower than “3″ represented *intoleran*ce or *low tolerance* and a score of 3 or higher represented (*high) tolerance*. The data was then descriptively and inferentially analysed into tables using frequencies, percentages and cross-tabulations, as well as Pearson Chi-Square tests to determine the relationship between tolerance and socio-demographic factors. All computations were done using SPSS version 25.

It is worth noting that owing to differences in the social stratification of the societies where the two institutions are situated, race and ethnicity are used interchangeably as proxy indicators for culture to allow for easy comparability of data. Also, because the questionnaires were anonymous and the sensitive nature of the questions, some respondents may have been reluctant to respond to some of the items, which led to missing values in some of the variables. There is also a strong likelihood of social desirability in the responses. Finally, because the data was collected from only two public universities, its generalizability to the larger high education sector in the respective countries is limited. Limitations notwithstanding, the trends are substantively and theoretically important—the instrument can be adopted or adapted for use in higher samples or other contexts.

## Ethics Statement

The ethics approval was obtained from the Faculty of Health and Social Sciences of the North-West University (Mafikeng Campus). Participation was voluntary and anonymous; data was treated with optimum confidentiality.

## CRediT Author Statement

**Elizabeth Biney:** Conceptualization; Investigation; Writing - original draft. **Olusegun Sunday Ewemooje:** Methodology; Formal analysis. **Acheampong Yaw Amoateng:** Resources; Writing - review & editing.

## CRediT authorship contribution statement

**Elizabeth Biney:** . **Olusegun Sunday Ewemooje:** . **Acheampong Yaw Amoateng:** Supervision.

## Declaration of Competing Interest

None.

## Appendix Supplementary materials

Image, application 1

Image, application 2

Image, application 3

Image, application 4
