# Supplementary material for: Comparative survey data on sociodemographic predictors of diversity tolerance among selected university students in Ghana and South Africa
Source: Data Brief. 2021 Jan 17;34:106771. doi: 10.1016/j.dib.2021.106771 (PMC7820931; doi:10.1016/j.dib.2021.106771)
Supplement: Supplementary file 3 [file mmc3.docx]

**TOLERANCE TO DIVERSITY AT THE UNIVERSITY OF GHANA, LEGON, GHANA**

**UNIVERSITY OF GHANA**

| Respondent’s name | |  | | | | | | | | | | | | Interview time |
| --- | --- | --- | --- | --- | --- | --- | --- | --- | --- | --- | --- | --- | --- | --- |
| Respondent’s cell number | |  |  | |  |  |  |  |  | |  |  |  |  |
| Interviewer number |  |  | | Checked | |  | | Back checked | |  | | | | Date |

1. **How old are you? ***(Record age in complete years)*:**
2. **Are you male or female?**

| Male | 1 | Female | 2 |
| --- | --- | --- | --- |

1. **Which type of school did you attend for Senior Secondary School? ***Single response***

| Public school in urban area | 1 |
| --- | --- |
| Public school in rural area | 2 |
| Private school | 3 |
| Other (please specify) | 4 |

1. *****Ask participant’s ethnicity***

| Akan | 1 |
| --- | --- |
| Ga-Adangbe | 2 |
| Ewe | 3 |
| Other: (specify)................................... | 4 |

1. **What is your nationality? ***Single response***

| Ghanaian | 1 |
| --- | --- |
| Other West African countries | 2 |
| Rest of Africa | 3 |
| Other: Specify | 4 |

1. **What is your academic year of study? ***Single response***

| First year | 1 |
| --- | --- |
| Second year | 2 |
| Third year | 3 |
| Fourth year | 4 |

1. **What was your first year of registration for the above qualification?**

| 2 | 0 |  |  |
| --- | --- | --- | --- |

1. **In which College are you registered as a student? ***Single response***

| College of Humanities/Social Sciences | 1 |
| --- | --- |
| College of Basic and Applied Sciences (Agriculture/Veterinary Sciences/Biological/Physical Sciences) | 2 |
| College of Education/Extra Mural/Distance Education | 3 |
| College of Health Science (Medicine/Clinical Sciences | 4 |
| Other (specify) | 5 |

1. **Which of the following applies to you? In the semester the AVERAGE mark for ALL my subjects was... ***Single response***

| Below 40% | 1 |
| --- | --- |
| 40%-49% | 2 |
| 50%-59% | 3 |
| 60%-69% | 4 |
| 70%-74% | 5 |
| 75% or more | 6 |

**10. What language do you (MOSTLY) PREDOMINANTLY SPEAK at home? ONE ANSWER ONLY**

| English | 1 |
| --- | --- |
| Twi | 2 |
| Ga | 3 |
| Ewe | 4 |
| Mole/Dagbani | 5 |
| Hausa | 6 |
| Other African Language specify: | 7 |
| Other European or non-African language specify: | 8 |

**11. Please indicate the highest level of your parents’/guardians’ education**

|  | Father/ Guardian | Mother/ Guardian |
| --- | --- | --- |
| None/ No education | 1 | 1 |
| Primary education (some or complete) | 2 | 2 |
| Some secondary education but not completed | 3 | 3 |
| Completed secondary school (SSS/A’level) | 4 | 4 |
| A college diploma | 5 | 5 |
| A first degree | 6 | 6 |
| A postgraduate degree | 7 | 7 |

**12. At home, which parents or guardians do you live with?**

| Both my mother and father in the same household | 1 |
| --- | --- |
| Only my mother | 2 |
| My mother and stepfather | 3 |
| Only my father | 4 |
| My father and stepmother | 5 |
| Some of the time in my mother’s home and some in my father’s home | 6 |
| Other relatives (aunt, uncle, grandparent) | 7 |
| Guardian/foster parent who is not a relative | 8 |
| No parents or guardians (I live alone) | 9 |

**13. How would you describe your family’s socioeconomic status compared to other families in the area where you live?**

| We are richer than most | 1 |
| --- | --- |
| We are poorer than most | 2 |
| We have about the same amount of money as most | 3 |

**14. What is your family’s religious affiliation? ***Single response***

|  | Self | Mother/Guardian | Fathe/Guardianr |
| --- | --- | --- | --- |
| Christian | 1 | 1 | 1 |
| Muslim | 2 | 2 | 2 |
| Traditional African | 3 | 3 | 3 |
| Other: Specify (specify)......................................... | 4 | 4 | 4 |

**15. If affiliation is Protestant, which Denomination?**

|  | Self | Mother | Father | Guardian |
| --- | --- | --- | --- | --- |
| Anglican | 01 | 01 | 01 | 01 |
| Lutheran | 02 | 02 | 02 | 02 |
| Methodist | 03 | 03 | 03 | 03 |
| Presbyterian | 04 | 04 | 04 | 04 |
| Baptist | 05 | 05 | 05 | 05 |
| Quaker/Friends | 06 | 06 | 06 | 06 |
| Mennonite | 07 | 07 | 07 | 07 |
| Dutch Reformed | 08 | 08 | 08 | 08 |
| Calvinist | 09 | 09 | 09 | 09 |
| Evangelical | 10 | 10 | 10 | 10 |
| Pentecostal | 11 | 11 | 11 | 11 |
| Independent | 12 | 12 | 12 | 12 |
| Church of Christ | 13 | 13 | 13 | 13 |
| Zionist Christian Church | 14 | 14 | 14 | 14 |
| Jehovah’s Witness | 15 | 15 | 15 | 15 |
| Seventh Day Adventist | 16 | 16 | 16 | 16 |
| Mormon | 17 | 17 | 17 | 17 |

**16. How often do you attend church/mosque/synagogue/temple?**

| Never | 0 |
| --- | --- |
| Only on special occasions | 1 |
| Occasionally (several times a year) | 2 |
| Frequent attendance | 3 |
| Fairly Regular (Almost weekly) | 4 |
| Regular (Weekly) | 6 |

**17. How often does your family members (Father/Mother/siblings attend church / mosque / synagogue / temple etc.?**

| Never | 0 |
| --- | --- |
| Only on special occasions | 1 |
| Occasionally (several times a year) | 2 |
| Frequent attendance | 3 |
| Fairly Regular (Almost weekly) | 4 |
| Regular (Weekly) | 6 |

**18. How religious do you consider yourself to be?**

| Not religious at all | 1 |
| --- | --- |
| Somewhat religious | 2 |
| Moderately religious | 3 |
| Very religious | 4 |
| Extremely religious | 5 |

**19. How important is religion in your life?**

| Not at all important | 1 |
| --- | --- |
| Not very important | 2 |
| Somewhat important | 3 |
| Very important | 4 |
| Extremely important | 5 |
| Refused to answer | 8 |
| Don’t know (DNR) | 9 |

**20. To what extent do you agree with the following statements?**

|  | **Strongly**  **disagree** | **Disagree** | **Neutral** | **Agree** | **Strongly agree** |
| --- | --- | --- | --- | --- | --- |
| a. Foreigners should be allowed into Ghana if they can make a contribution | 1 | 2 | 3 | 4 | 5 |
| b. I would have a higher standard of living if there were no foreigners living in this country | 1 | 2 | 3 | 4 | 5 |
| c. I think foreigners should be given the same opportunities as local citizens | 1 | 2 | 3 | 4 | 5 |
| d. I would date a foreigner | 1 | 2 | 3 | 4 | 5 |
| e. Attacks against foreigners are wrong | 1 | 2 | 3 | 4 | 5 |
| f. Foreigners tend to increase the crime rate in Ghana | 1 | 2 | 3 | 4 | 5 |
| g. Foreigners take jobs away from Ghanaians | 1 | 2 | 3 | 4 | 5 |
| h. Ghana is letting in too many foreigners | 1 | 2 | 3 | 4 | 5 |
| i. foreigners bring in the possibility of disease | 1 | 2 | 3 | 4 | 5 |

**21. How much do you trust each of the following types of people?**

|  | Don’t trust at all | Distrust slightly | Neutral | Trust slightly | Trust fully |
| --- | --- | --- | --- | --- | --- |
| Your relatives | 1 | 2 | 3 | 4 | 5 |
| Other Ghanaians | 1 | 2 | 3 | 4 | 5 |
| West Africans | 1 | 2 | 3 | 4 | 5 |
| Other Africans | 1 | 2 | 3 | 4 | 5 |

**22 To what extent do you feel comfortable about doing the following with somebody of a different ethnicity than your own? ***One response per row***

|  | **To no extent** | **To a small extent** | **To a medium extent** | **To a large extent** | **To a very large extent** |
| --- | --- | --- | --- | --- | --- |
| Attending lectures | 1 | 2 | 3 | 4 | 5 |
| Participating in a study group | 1 | 2 | 3 | 4 | 5 |
| Sharing accommodation but not the same room | 1 | 2 | 3 | 4 | 5 |
| Sharing a room | 1 | 2 | 3 | 4 | 5 |
| Being friends | 1 | 2 | 3 | 4 | 5 |
| Dating | 1 | 2 | 3 | 4 | 5 |
| Socialising with people from another ethnic group | 1 | 2 | 3 | 4 | 5 |
| Having friends who are members of a different ethnic group | 1 | 2 | 3 | 4 | 5 |

**23. How do you feel about the following statements? ***One response per row***

|  | **Strongly Disagree** | **Disagree** | **Neutral** | **Agree** | **Strongly Agree** |
| --- | --- | --- | --- | --- | --- |
| It is okay for people from different ethnic groups to attend social functions (parties, weddings, funerals etc.) together. | 1 | 2 | 3 | 4 | 5 |
| It is okay to associate mostly with your own ethnic group at a mixed social function. | 1 | 2 | 3 | 4 | 5 |
| It is okay for people from different ethnic groups to date each other. | 1 | 2 | 3 | 4 | 5 |
| People should be free to marry whoever they want to marry regardless of their ethnicity. | 1 | 2 | 3 | 4 | 5 |

**THANK YOU FOR YOUR COOPERATION**
